# Connectivity as a Mediating Mechanism in the Cybervictimization Process

**DOI:** 10.3390/ijerph17124567

**Published:** 2020-06-24

**Authors:** Isabel Cuadrado-Gordillo, Inmaculada Fernández-Antelo

**Affiliations:** Department Psychology and Anthropology, Faculty of Education, University of Extremadura, 06071 Badajoz, Spain; iferant@unex.es

**Keywords:** social connectivity, cybervictimization, adolescent, social identity, self-esteem

## Abstract

This paper explores the relationship between social connectivity and cybervictimization as it is mediated by psychosocial variables such as social identity and self-esteem. Likewise, it analyses the moderating role in that relationship played by adolescents’ perception of cyberbullying. The sample consisted of 2072 adolescents (48.7% girls) aged between 14 and 18 (Mean = 15.78, Standard Deviation = 1.02) years. Through the use of five questionnaires, an explanatory model is constructed that shows the direct and indirect relationships between the factors analysed, the predictive values that social connectivity can reach when applied to the virtual environment, and the perception of cyberbullying in the victimization processes. The results indicate that self-esteem and social identity are protective factors in the establishment of healthy virtual relationships and avoidance of cybervictimization situations. Moreover, the equation of cyberbullying with aggressive or maladaptive styles of humour has an indirect influence on the link between connectivity and cybervictimization.

## 1. Introduction

The phenomenon of cyberbullying is a public health problem that affects not just children and adolescents but adults as well, and its comprehension and intervention require a multidimensional approach. One of the difficulties that researchers encounter when analysing the prevalence of cyberbullying and establishing its interrelationships with other variables (psychological, social, emotional, familial, etc.) is the perception that children and adolescents have of it. The criteria they use to classify aggressive cyber behaviour as episodes of cyberbullying have been explored in many studies over the last decade [1,2,3]. The results have revealed that the adolescents’ interpretation of this phenomenon differs considerably from that used by researchers so that very different descriptions are given of the same reality [4]. While researchers widely resort to five criteria that define cyberbullying (intent to harm, power imbalance, repetition, publicity, and anonymity) [3], adolescents only use some of these, establishing first- and second-order relationships between them [5], and incorporate others such as revenge [6]. Thus, [7] and [1] note that, as a priority, young Europeans resort to two criteria to define and identify cyberbullying situations: intent to harm and power imbalance. Other researchers, however, note mainly intentionality [8], the public dimension of cyberaggression [9,10], or introduce other criteria such as the impact on the victim that the aggression might have [11]. More recent studies [12] have explored adolescents’ possible perceptual structures about cyberbullying, revealing a network of principal and subsidiary relationships among the different criteria that make up this phenomenon, and warning that these structures may vary depending on whether the role played is that of aggressor or victim. It has also been noted that some cyberbullying modalities are not interpreted as such by adolescents but rather as patterns of social interaction typical of their age [13,14], with this being evidence for a process of normalization of aggression that many adolescents are incorporating into their forms of interpersonal relationships.

Other work has focused efforts on detecting the possible variables that may explain the modification of these perceptual structures, as well as on a search for factors that are predictors, mediators, and moderators of cyberbullying. In this sense, there have been studies of adolescents’ need for affiliation and relationships in a virtual world in which they spend more and more time each day [4,15]. It is paradoxical, however, that in a world so interconnected as is the cyber-world many adolescents feel isolated and find serious difficulties in taking on social roles and responsibilities [16]. The online/offline duality in which they live their lives has meant that social behaviours which in other times would have been restricted to classroom settings now also occur in virtual environments. With the Internet, some of the social needs that cannot be satisfied in face-to-face interactions are being met through virtual social networks and platforms [17]. The problem arises when, in a virtual environment that has now become as important as that which is off-line, young people are unable to connect with their peers and maintain satisfactory social relationships [18]. This lack of social connectivity has negative repercussions for adolescents’ psychosocial health (due to the strong feeling of belonging that they manifest), repercussions that are associated with high levels of loneliness and social distress [19], low scores on self-esteem [16], depression, etc. [20]. Likewise, the difficulties in cybernetic social connectivity have been related to cyberbullying [21,22]. The lack of support systems and the psychological distress produced by feelings of isolation are strong predictors of cybervictimization [23]. However, as McLoughlin et al. [24] note, there has been very little research examining the relationship between social connectivity and cyberbullying.

Authors such as Lee and Robins [16] analyse the relationship of social connectivity with self-esteem and social identity in off-line contexts. The translation of these links to virtual environments has, however, received little attention. The case of social identity is of particular relevance because of the impact it has on how adolescents identify with other people or groups [25] and on how they face challenging situations such as victimization or a lack of social connectivity. As Côté [26] warns, people who feel excluded have greater difficulty in feeling identified with others and are therefore more likely to give up and not activate effective coping strategies. In this sense, victims’ lack of social connectivity constitutes a risk factor not only because they have no support network available but also because it acts against their construction of a solid and healthy identity [27]. Although the cyber-world offers the possibility of forging new identities and overcoming difficulties that may be experienced in physical contexts, the person’s identity capital will be eroded if the situations of exclusion are themselves transferred to virtual environments.

The search for social support also implies the search for a desirable psychological state, with optimal levels of self-esteem. In the last decade, adolescents’ participation in virtual social network platforms has grown exponentially, causing many of them to become addicted to Internet activities [28]. The reasons adolescents use these platforms include the feeling of belonging and connectivity, appeasing negative emotions, and coping with depression [17]. Sometimes, however, the effects actually achieved are contrary to those which are desired, and the level of self-esteem decreases due, among other factors, to the volatility and superficiality of the social relationships that are created on virtual platforms, to the affiliation with people who are depressed, or to the exclusion and isolation experienced on these platforms [29,30].

Previous research shows that social connectivity, self-esteem, and social identity are variables that are interrelated in face-to-face contexts [16,31]. Such evidence is less clear, however, when the environment is cybernetic. Similarly, it is difficult to determine whether the influence of these variables on cybervictimization processes is stronger when they act interrelatedly or in isolation. Previous studies treat each of these variables individually without linking their effects to the cyberbullying phenomenon. The present study addresses these challenges in pursuing the following objectives: (i) to explore the relationship between connectivity and cybervictimization, (ii) to analyse the mediating effect of self-esteem and social identity on the relationship between connectivity and cybervictimization, and (iii) to determine the moderating role that the perception of cyberbullying plays in the relationship between connectivity and cybervictimization.

## 2. Methods

### 2.1. Participants

The subjects were 2072 adolescents (48.7% girls) aged between 14 and 18 years (M = 15.78; SD = 1.02) selected by stratified probabilistic cluster sampling to cover different geographical areas (urban, peripheral, and rural) and socio-economic classes. The clusters chosen were the schools in which the subjects were studying, and, in these schools, a random selection was made of the courses from which participation would be requested. In total, 18 schools were selected, and 5–6 classrooms participated in each one. In schools there are approximately 3–4 classrooms of the same course. From each of the courses we randomly chose a classroom.

### 2.2. Instruments

To identify cybervictims and to explore the perception that adolescents have of cyberbullying, we applied the questionnaire presented in Cuadrado and Fernández [13]. Its first three items allow one to determine whether the participants have been victims, aggressors, or witnesses of cyberbullying episodes in the last two months, and how often they have suffered, committed, or observed these aggressions on a four-point scale (“never”, “once or twice”, “once a week”, and “several times a week”). The consideration of this period of time is due to the immediacy criterion that modulates the behaviours of adolescents, mainly those related to virtual scenarios. In this way, the delimitation of the frequency of the aggressions suffered, committed, or observed is facilitated to the participants. They also provide information about the kind of cyberaggression suffered, committed, or observed, grouped into eight types: insults (including homophobia), threats (including blackmail), spreading false rumours, exclusion (from contact lists, social networking, etc.), identity theft, sexting, posting denigrating images or videos, and recording and disseminating physical aggressions. In the following, we present by way of example the question that allows the adolescents who consider themselves to be victims of cyberbullying to be identified. They were told to indicate how often during the past 2 months they had suffered any of the following behaviours: (1) “I have been insulted through the mobile phone or Internet”; (2) “I have been threatened or blackmailed through the mobile phone or Internet”; (3) “Lies and false rumours have been spread about me through the mobile phone or Internet”; (4) “I have been removed from contact lists on social networks, group chats, or emails so as to exclude me”; (5) “I have had someone pretend to be me, and my email, private chat rooms, or social network profile have been accessed without my permission”; (6) “They have sent by mobile phone or Internet incriminating photos or videos, which are denigrating or demeaning to me”; (7) “They have recorded fights in which I participated and spread them through mobile phones, social networks, or other cyber means”; (8) “They have sent sexual or erotic type of content in which I took part”. A reliability analysis showed satisfactory internal consistency (Cronbach’s alpha: α_victimization_ = 0.87). More specifically, the internal consistency values for each of the abuse modalities range from 0.69 to 0.81. The remaining 40 items are aimed at exploring the perception the adolescents have of cyberbullying and the modalities in which it manifests itself. Implicit in these questions are the criteria of repetition, imbalance of power, intent to harm, publicity, anonymity, revenge, and harmless forms of social relationship. The participants have to respond to the questions on a 5-point Likert scale where 1 is “disagree” and 5 “strongly agree”. The level of internal consistency presented by this block of questions is α_perception_ = 0.76. An example of this type of questions is as follows: “Why do you think some peers threaten others through telephone calls?” (1) Because they do not dare do it face to face for fear of reprisals; (2) Because they can hide their identify and inflict fear on others who are stronger; (3) Because it is the way they have of relating; (4) Because that way they feel more powerful; (5) Because it the only way they have to get what they want; (6) Because they feel more accepted by their friends; (7) Because it is a way of getting revenge; (8) Because they record the telephone calls and then spread them so that the victim repeatedly feels fear; (9) Because they like to see how others suffer; (10) They are jokes or other ways of having fun that are typical of adolescents”.

The value of social connectivity was calculated using the Social Connectedness Scale—Revised [19]. This consists of 20 items that measure the feeling of connection with others in the respondent’s social environment by means of a 6-point Likert scale (1 strongly disagree—6 strongly agree). This scale has been used with adolescents in online contexts [32,33]. The reliability value reached in the present study was α= 0.88.

The level of self-esteem was measured using the Rosenberg Self-Esteem Scale [34]. This scale evaluates individual self-esteem using 10 items related to self-respect and self-acceptance. The responses are measured on a 4-point scale where 1 corresponds to strongly disagree and 4 corresponds to strongly agree. The validation results showed the existence of two factors: positive self-esteem (6 items) and negative self-esteem (4 items), with an adequate value of internal consistency being reached in the present study (α_positive self-esteem_ = 0.79; α_negative self-esteem_ = 0.75).

The final questionnaire used was the Three-Dimensional Strength of Group Identification Scale [35]. This consists of 12 items whose responses are scored on a 7-point Likert scale (1 strongly disagree—7 strongly agree). Each of three dimensions is measured by 4 items: cognitive centrality (α = 0.82), in-group affect (α = 0.80), and in-group ties (α= 0.80).

### 2.3. Procedure

The study was approved by the Ethics Committee of University of Extremadura (Ref: 18/2017). Because most of the participants in this study were underage, it was necessary to obtain consent from their parents to collect the data, while guaranteeing confidentiality and anonymity of the participants. It was also important to have the approval of the Education Administration to access the schools and to hand out the questionnaires. Once both forms of consent had been obtained, the researchers went to the different schools and, thanks to the collaboration of their management teams, accessed the different classrooms to distribute the questionnaires. Of each classroom, 18 to 24 adolescents participated. The researchers remained in the classrooms until the questionnaires had been completed in order to clarify any possible doubts. The instrument battery had to be answered at the researcher’s visit, granting a time that ranged from 60 to 75 min. Younger participants used 75 min while older participants took less than 60 min.

### 2.4. Analysis

The analyses were carried out in four phases. In the first, the participants’ perception of cyberbullying is explored through a factor analysis. In this phase, the scores obtained for the criterion “form of social relationship” are used to analyse the moderating effect. In the second phase, the correlations of all the variables are calculated. The software used in both phases is SPSS 23.0 (SPSS Inc., Chicago, IL, USA). In the third, the mediation analysis is performed using the PROCESS macro for SPSS [36] to determine whether the influence of social connectivity on cybervictimization is mediated by psychosocial variables such as self-esteem and social identity. The fourth phase explores the possible moderating effects that the perception of cyberbullying (which in this study will be identified with the name of normalization of cyberbullying) might have on the relationship between social connectivity and cybervictimization. The PROCESS macro provides 95% bias-corrected bootstrap confidence intervals for the indirect effects from 1000 re-samples.

## 3. Results

### 3.1. Preliminary Analyses

The results of the factor analysis reveal, on the one hand, the underlying domains that are present in adolescents’ perception of cyberbullying and, on the other hand, the factor scores also inform of the importance that they assign to each one of them. The results reveal that adolescents fundamentally resort to three criteria to identify cyber-aggressions as cyberbullying episodes: intent to harm (component 1), publicity of the aggressions (component 2), and power imbalance (component 4) (Table 1), relegating to a second level other criteria such as the anonymity of the aggressor (component 5) and the repetition of the aggressive behaviour (component 7). However, the results also show that many adolescents normalize aggressive behaviours and interpret them as harmless mechanisms and forms of social relationship that are characteristic of adolescence (component 3) (Table 1). They understand that spreading offensive messages from others, threatening or insulting them, among other behaviours, are not aggressive behaviours but rather harmless guidelines for social interaction. The scores obtained on this factor constitute the variable normalization of cyberbullying. The criterion revenge (component 6) also appears in the results. Although it is not used by researchers, the adolescents give it a residual value.

The correlation analysis reveals that all the dependent variables are negatively associated with cybervictimization (Table 2), which suggests that social connectivity (*r* = −0.53, *p*< 0.001), self-esteem (*r* = −0.39, *p* < 0.001), social identity (*r* = −0.29, *p* < 0.01), and a playful perception of cyberbullying (*r* = −0.47, *p* < 0.001) are protective factors against the likelihood of becoming a cybervictim. Another result that stands out is that this perception of cyberbullying as a form of social relationship is not linked to the variables self-esteem and social identity.

### 3.2. Self-Esteem and Social Identity as Mediating Variables

With the mediation analysis, the aim was to see whether, on the one hand, social connectivity is a predictor of cybervictimization and, on the other, self-esteem and social identity mediate the relationship between social connectivity and cybervictimization. The results indicate a significant negative association between connectivity and cybervictimization (β = −0.29, *p* < 0.001) (Model 1 of Table 3). Likewise, social connectivity was found to be a strong predictor of self-esteem (β = 0.33, *p* < 0.001) (Model 2 of Table 3), and in exerting control over social connectivity, self-esteem is significantly negatively associated with cybervictimization (β = −0.27, *p* < 0.001) (Model 4 of Table 3). Moreover, it is found that social connectivity can predict social identity (β = 0.26, *p* < 0.01) (Model 3 of Table 3) and that this is a predictor of cybervictimization (β = −0.25, *p* < 0.01) (Model 4 of Table 3). Finally, there is a direct relationship between social connectivity and cybervictimization (β = −0.21, p < 0.05) (Model 4 of Table 3).

The bias-corrected percentile bootstrap method indicates that the indirect effect of social connectivity on cybervictimization via self-esteem is significant (*ab* = −0.18, *p* < 0.05, 95% CI = [−0.11/−0.24]). Similarly, the indirect effect of social connectivity on cybervictimization via social identity is also significant (*ab* = −0.16, *p* < 0.05, 95% CI = [−0.07/−0.20]).

### 3.3. Mediated Moderation between Social Connectivity and Cybervictimization

The normalization of cyberbullying exerts a moderating effect both on the relationship between social connectivity and cybervictimization as well as on the mediating relationship that self-esteem and social identity establish between connectivity and cybervictimization.

The results show a significant association of social connectivity with cybervictimization (β = −0.26, p < 0.001) that is moderated by the variable normalization of cyberbullying (β = −0.35, *p* < 0.001) (Model 1 of Table 4). Simple slope tests indicate that, at high levels of perception of cyberbullying (1 SD above the mean), high levels of social connectivity are associated with low levels of cybervictimization (β_simple_ = −0.23, *p* < 0.01). At low levels of perception of cyberbullying (1 SD below the mean), however, the relationship between social connectivity and cybervictimization is not significant.

Model 2 indicates that the effect of social connectivity on self-esteem is significant (β = 0.28, *p* < 0.001) (Model 2 of Table 4), and that the normalization of cyberbullying does not have any moderating effect on that relationship (β = 0.11, *p*>0.05). In contrast, the significant effect of social connectivity on social identity (β = 0.20, *p* < 0.01) is moderated by the normalization of cyberbullying (β = 0.22, *p* < 0.01) (Model 3 of Table 4). Simple slope tests show that when there are high levels of normalization of cyberbullying (1 SD above the mean), high levels of connectivity are related to high levels of social identity (β_simple_ = 0.27, *p* < 0.001). When there are low levels of normalization of cyberbullying, this relationship is weaker (β_simple_= 0.19, *p*< 0.05). Finally, Model 4 reveals that both self-esteem (β = −0.25, *p* < 0.001) and social identity (β = −0.19, *p* < 0.01) are significantly related to cybervictimization. Similarly, it shows that the normalization of cyberbullying has a moderating effect on the relationships between self-esteem and cybervictimization (β = −0.20, *p* < 0.01) and between social identity and cybervictimization (β = −0.17, *p* < 0.05). Simple slope tests indicate that when there are high levels of normalization of cyberbullying, high levels of self-esteem are related to low levels of cybervictimization (β_simple_ = −0.33, *p* < 0.001) and that when there are low levels of normalization of cyberbullying, high levels of self-esteem are related to low levels of cybervictimization but to a lesser extent (β_simple_= −0.21, *p* < 0.01). When there is a high level of normalization of cyberbullying, high levels of social identity are related to low levels of cybervictimization (β_simple_ = −0.28, *p* < 0.001). Low normalization of cyberbullying reveals that high social identity is related to low cybervictimization (β_simple_ = −0.20, *p* < 0.01).

The results of the bias-corrected percentile bootstrap confirm that the normalization of cyberbullying significantly moderates the indirect effect of social connectivity on cybervictimization. The indirect effect was significant for both high levels (*ab* = −0.21, *p*< 0.01, 95% CI = [0.14/0.27]) and low levels (*ab* = −0.18, *p* < 0.05, 95% CI = [−0.11/−0.22]) of normalization.

## 4. Discussion

The results of this study show that social connectivity has an indirect impact on cybervictimization through psychosocial variables such as self-esteem and social identity. Previous studies confirm this relationship in noting that high levels of social connectivity can generate more positive mental health and well-being [37] and can predict the activation of coping strategies in response to cybervictimization [24]. All this suggests that these psychosocial variables may help explain why adolescents who have difficulties relating to and being connected with their peers are more likely to become cybervictims, and conversely, how self-esteem and social identity could be used as protective mechanisms to avoid being cybervictimized.

The importance for adolescents to feel accepted and integrated into a social group has endowed virtual platforms with unusual power in the way that young people relate to each other on them. The virtual environment is no longer to be understood as a context of social compensation for isolated people or people with little ability to relate to their peers in off-line settings [38]. At present, when the popularity of a person is measured on the basis of the number of followers they have on many virtual platforms, the number of “likes” they obtain, or the volume of comments they receive from people (most of whom are strangers), the cyber-world can become a space of exclusion and isolation [39]. Despite the number of hours that adolescents spend connected to the Internet viewing the comments, photos, videos, and other material that they and their peers upload, many of them feel lonely [38]. This is the scenario in which young people have to face the difficult task of building a strong and healthy social identity that will allow them to identify themselves as individual beings within a community [40]. The results of the present study show that there is a relationship between social connectivity and cybervictimization that is mediated by social identity. In other words, those who can count on a secure and satisfactory social network tend to present a more constructive social identity where they feel loved, accepted, and respected by others and which in turn allows them to feel that they have the strength to face cyberbullying situations and not become victims. According to Pegg et al. [15], the construction of online social identity has enormous potential to influence thought, emotion, and behaviour. Moreover, insofar as adolescents confront, explore, and seek to understand their place in the world through this network of connections, social identity plays a significant role in presenting and defining acceptable or prescribed group norms and behaviours [41]. These results are also in line with those of previous research which has noted a relationship between social connection and cybervictimization mediated by psychological variables that influence mental health [22,42]. One of these variables is social identity, which could act either as a protective factor against cyberaggressive situations or as an enhancer of psychosocial distress and a facilitator of victimization.

Another mediating factor in the relationship between social connectivity and cybervictimization is self-esteem. Feeling valued by oneself within a social group favours the construction of more sustainable and healthy support networks, as well as being a protective factor against the threat of experiencing situations of cybervictimization. As noted by [43,44], while low self-esteem is a predictor of cybervictimization, there is also—as was seen in the indirect effects of the present study—an inverse relationship in that experiencing victimization implies a decline in the levels of self-esteem. With regard to the relationship between self-esteem and social connectivity, our results are in line with those of Valkenburg et al. [45] who state that young people’s self-esteem exerts a marked influence on the use they make of online social platforms. However, our results also suggest an inverse relationship confirming those of previous studies which indicate that the satisfaction adolescents get from interacting on these virtual platforms and receiving positive comments has great benefits for their self-esteem [46]. In short, the subjective well-being provided by these protective factors allows young people to regulate their social experiences and adopt healthier interpersonal forms of behaviour [31].

Additionally, the results of the present study show the perception that adolescents have of cyberbullying and how many of them interpret cyberaggressive behaviour as apparently harmless patterns of social relationship and communication mechanisms typical of adolescence. There is therefore a tendency for adolescents in a position as witnesses or aggressors to downplay and normalize aggressive behaviour. But even those in the role of victim show signs of normalizing abusive behaviour, and, as Cuadrado and Fernández [47] point out, in such cases, they are forced to resort to all kinds of moral justifications so as not to see their self-esteem threatened. This distorted perception of cyberbullying in turn plays a role as regulator between connectivity and cybervictimization, a reflection of the complex network of relationships established between the variables that were analysed here. Specifically, the present results show the normalization of violence as being a possible factor that would diminish the experimentation of cybervictimization situations. Adolescents’ interpretation of cyberaggression as being just a playful mechanism of social relationship constitutes a defensive framework that they use to protect themselves and not feel offended or attacked. There are also studies pointing in this direction such as those of Steer et al. [48] who analyse the variables that cause adolescents to interpret certain behaviours as sometimes being just jokes and sometimes being situations of aggression.

Furthermore, the present results reveal that, via self-esteem and social identity, the normalization of cyberbullying has a moderated mediatory effect on the relationship between connectivity and cybervictimization. When adolescents interpret cyberbullying as just playful patterns of social relationship, this conditions both their social connectivity and the process of their construction of an identity. It also affects positively their self-esteem, which would contribute to a decrease in the experimentation of cyber-aggressions. The adoption of maladaptive and even aggressive styles of humour that lead to the normalization of violence may be motivated by a desire to maintain online social relationships and avoid being excluded [14,49], thus reflecting the influence of group norms and behaviours in the formation of individual identity [15]. These styles of humour could promote episodes of cyberbullying, not only for the role of aggressor [50,51] but also for that of victim. Self-esteem could play a protective role in this relationship between the normalization of violence and cybervictimization, protecting both the individual’s subjective well-being [52] and their ability to cope with abusive situations [53].

## 5. Conclusions

In a world where we are ever more dependent on technology and where we are accepting new virtual forms of peer-to-peer interaction and relationship, questions are arising about adolescents’ social connectivity and sense of belonging that require responses which take the cyber-world to be an important social environment. The problems of cyberbullying have to be added to those of addiction to online social platforms that are being detected in young people. The results of this study have shown how complex the network of relationships that associate social connectivity with cybervictimizationis, with the adolescents’ self-esteem and social identity playing a mediating role. Although previous studies have investigated some of these links in off-line contexts, many questions have remained open when this context becomes virtual. This in particular has been one of the main contributions of the present study. Another contribution lies in associating how adolescents perceive cyberbullying with the set of interactions that are established between social connectivity and cybervictimization. The result confirms the multidimensional nature of the cyberbullying phenomenon and reveals the influences that moderate and mediate in the relationship between the different variables that were analysed. The knowledge deriving from this study will aid in allowing a better approach to understanding the processes of victimization, while providing keys to designing programs of prevention and intervention concerning cyberbullying.

### Limitations

The main limitation found in this study is the transversality of its data. As a longitudinal study cannot be carried out, it is difficult to draw causal conclusions and determine predictive indicators of cybervictimization. The results of this research should be interpreted as a detailed exploration of the network of relationships that could be established between cybervictimization and a set of psychosocial variables, as well as a starting point for the development of longitudinal studies that facilitate the verification of protective and predictive factors of cyberbullying.

## Figures and Tables

**Table 1:** Total variance explained by the components.

Component	Initial Eigenvalues			Extraction Sums of Squared Loadings		
	Total	% of Variance	Cumulative %	Total	% of Variance	Cumulative %
1	4.26	31.25	31.25	4.26	31.25	31.25
2	2.78	20.06	51.31	2.78	20.06	51.31
3	1.97	15.99	67.30	1.97	15.99	67.30
4	1.68	12.54	79.84	1.68	12.54	79.84
5	0.77	8.48	88.32			
6	0.53	6.65	94.97			
7	0.45	5.03	100			

Extraction method: Principal component analysis.

**Table 2:** Correlations of all the study’s variables.

Variables	1	2	3	4	5	6
Gender	-					
Social connectedness	0.26 **	-				
Self-esteem	0.19 *	0.38 ***	-			
Social identity	0.08	0.31 **	0.33 **	-		
Cyberbullying normalization	0.20 *	0.23 *	0.11	0.14	-	
Cybervictimization	0.21 *	−0.53 ***	−0.39 ***	−0.29 **	−0.47 ***	-

* *p* < 0.05; ** *p* < 0.01; *** *p* < 0.001.

**Table 3:** Testing the mediation effect of social connectivity on cybervictimization.

Predictors	Model 1		Model 2		Model 3		Model 4	
	Cybervictimization		Self-esteem		Social Identity		Cybervictimization	
	*β*	*t*	*β*	*t*	*β*	*T*	*β*	*t*
Gender	0.04	2.39	0.09	3.08	0.11	3.56	0.07	2.87
Social connectedness	−0.29	−0.8.74 ***	0.33	8.98***	0.26	8.09 **	−0.21	6.14 *
Self-esteem							−0.27	8.29 ***
Social identity							−0.25	7.99 **
*R^2^*	0.21		0.24		0.25		0.48	
*Adj. R^2^*	0.17		0.19		0.19		0.41	
*F*	33.79 ***		38.45 ***		36.74 ***		54.87 ***	

* *p* < 0.05; ** *p* < 0.01; *** *p* < 0.001.

**Table 4:** Coefficients of the moderated mediation model.

Predictors	Model 1		Model 2		Model 3	
	Cybervictimization		Self-Esteem		Social Identity	
	*β*	*t*	*β*	*t*	*β*	*T*
Gender	0.03	2.19	0.06	2.67	0.14	3.81
Social connectedness (CS)	−0.26	−8.25 ***	0.28	8.76 ***	0.29	8.97 ***
Normalization cyberbullying (NC)	−0.35	−10.77 ***	0.09	3.01	0.20	6.76 **
CS × NC	0.21	7.14 **	0.11	3.65	0.22	7.32 **
Self-esteem (SE)						
Social identity (SI)						
SE × NC						
ID × NC						
*R^2^*	0.29		0.27		0.31	
*Adj. R^2^*	0.20		0.20		0.28	
*F*	34.21 ***		33.87 ***		38.62 ***	

* *p* < 0.05; ** *p* < 0.01; *** *p* < 0.001.
